# Expression of cell adhesion molecule CD44 in mucoepidermoid carcinoma and its association with the tumor behavior

**DOI:** 10.1186/s13005-016-0102-4

**Published:** 2016-01-29

**Authors:** Nada Binmadi, Azza Elsissi, Nadia Elsissi

**Affiliations:** Department of Oral Diagnostic Sciences, King Abdulaziz University, P.O. Box: 80200, Jeddah, 21589 Saudi Arabia; Oral Pathology Department, University of Mansoura, Mansoura, Egypt

**Keywords:** Salivary gland tumor, Mucoepidermoid carcinoma, CD44, Adhesion molecules

## Abstract

**Background:**

The most common malignant salivary gland tumors that affect both adult and children is mucoepidermoid carcinoma. It usually affects both minor and major salivary glands but parotid gland is considering the most common site in which this tumor arises. CD44, a trans-membrane glycoprotein, is an adhesion molecule of cell surface that play a role in the connections between cell-cell and cell-matrix. Many malignant tumors express high levels of CD44, thus, CD44 may be used as an indicator of aggressive behavior of some human malignancy. We evaluate CD44 expression in different grades of mucoepidermoid carcinoma and determine whether expression of CD44 can be used to predict tumor aggressiveness.

**Methods:**

Fifteen cases of mucoepidermoid carcinoma were retrieved from the oral pathology archives and grouped according to the histological grade as well as the clinical behavior regarding metastases and/or recurrence. Tissue sections were immunohistochemically stained for CD44. CD44 staining was scored for intensity and proportion of cells stained.

**Results:**

A higher proportion of high-grade tumor tissues showed moderate or strong CD44 staining compared to low-grade tumors. Additionally, CD44 expression was stronger in tumors from patients with recurrences or metastases, but theses differences were not statistically significant.

**Conclusion:**

Our result showed that mucoepidermoid carcinomas are immunohistochemistry positive to CD44 compare to normal. A trend of CD44 expression associated with different histological grading and aggressive behavior of this tumor.

## Background

The most common type of malignant salivary gland tumor affecting both adults and children is mucoepidermoid carcinoma (MEC). It usually affects both minor and major salivary glands, but the parotid gland is considered the most common site at which this tumor arises and usually has better prognosis than other major salivary glands. Histologically, MECs are composed of epidermoid, mucous, and intermediate cells and are graded low, intermediate, or high according to one of the following grading systems: the modified Healey system, the Armed Forces Institute of Pathology (AFIP) grading system, and the Brandwien system. These grading schemes for MEC are important to determine tumor progression and patient management [1, 2].

CD44, a trans-membrane glycoprotein, is a cell surface adhesion molecule that plays a role in cell-cell and cell-matrix interactions. Many malignant tumors, such as head and neck, breast, and prostate cancer, express high levels of CD44 or its variants [3–5]. Thus, CD44 may be used as an indicator of aggressive behavior of some human malignancies. However, the role of CD44 in MEC remains unclear.

CD44 is encoded by a single gene located on the short arm of chromosome 11 in humans and is required to maintain complex tissue morphology. CD44 exists in a standard form (CD44s) and 10 distinct isoforms (CD44v) that arise from alternative splicing of mRNA and further posttranslational glycosylation [6]. The protein is composed of three domains: extracellular, transmembrane, and intracellular. Its extracellular portion contains the N-terminus, which primarily binds to the ligand hyaluronan (HA), where their interaction promotes signal transduction, regulates matrix assembly and cell migration, and maintains proliferation and differentiation of cancer stem cells (CSCs) [7–9]. CD44 is expressed in many normal cells such as lymphocytes, and epithelial and endothelial cells during hematopoiesis, embryonic development and wound healing [5].

Classical CD44 molecules are intimately involved in the pathogenesis of malignancies such as esophageal cancer, breast cancer, gastric cancer, and prostate cancer [4, 5, 7] CD44 has also been reported as a marker of CSCs in prostate, breast, and head and neck cancer and its expression in these tumors is associated with poor prognosis and aggressive behavior [4, 10, 11]. CSCs are stem-like cells that have the potential to regenerate a tumor mass and maintain their renewal ability. Given the important functions of CD44 in cell-cell and cell-matrix interactions, CSCs are usually postulated to play a vital role in cancer metastasis and aggressiveness.

MEC comprises 12–29 % of malignant tumors [1]. The histogenesis of salivary gland mucoepidermoid carcinoma still remains a controversial topic. Some believe that this tumor originates from myoepithelial cells, the excretory or the intercalated duct cells, or intermediate cells. Histologically, MECs are classified as low, intermediate, or high grade on the basis of the following features: the presence of cellular differentiation, cystic spaces, proportion of mucous cells, growth pattern, type of invasion, and cytological atypia. Many MECs were found to be positive for translocation of cyclic AMP response element-binding protein (CREB)-regulated transcription coactivator (CRTC1-MAML2) t(11;19) [12]. The prognosis of MEC depends mostly on the histological grade, since high-grade MEC is a highly aggressive tumor, while its low-grade variant usually demonstrates a more benign nature.

The treatment of choice in MEC is surgical resection. Whether adjunctive postoperative radiotherapy and/or chemotherapy improve the patient survival rate is not known because of the absence of a systemic analysis of published studies. Metastasis, which can affect patient survival, is a complex process and is not fully understood. We hypothesized that the destruction of adhesions by CD44 provides more free malignant cells that might act to facilitate recurrence and/or metastasis of MECs.

## Methods

Tissues from 15 patients with a specific diagnosis of mucoepidermoid carcinoma of the salivary gland from Mansoura University, Egypt, were collected from 2001 to 2006. The Ethics Committee of Mansoura University Hospital authorized the collection of specimens, and follow-up data was obtained from patients’ pathology reports. We followed the guidelines of human subjects in the Declaration of Helsinki. The hematoxylin and eosin-stained slides were reviewed and graded in accordance with the Brandwien grading system [13].

Immunohistochemical staining of tissue sections was conducted as follows. After dewaxing and hydration of the tissue sections, antigen retrieval was performed by microwaving in citrate buffer (10 mM citric acid, pH 6.0). Tissue sections were then blocked in 2 % bovine serum and incubated in a moist chamber at 4 °C overnight with anti-CD44 Std-antibody (Thermo Scientific, Clone 156-3C11). The slides were incubated for 10 min at 37 °C with a secondary biotinylated anti-mouse antibody and then incubated with streptavidin-HRP. Specimens were developed with DAB and the nucleus was counterstained with hematoxylin. The sections were photographed under a microscope and analyzed by two pathologists.

Semiquantitative evaluation was performed in tumor tissue using the Allred immunostaining scoring system [14]. The staining intensity was scored as 0 (none), 1 (mild), 2 (moderate), or 3 (strong); and the proportion of stained cells was score as 0 (none), 1 (>0–1 %), 2 (≥1–10 %), 3 (>10–33 %), 4 (>33–66 %) and 5 (>66–100 %). The sum of the proportion score and the intensity score was calculated to obtain a total score that ranged from 0 to 8. The significance of the differences between groups was evaluated using an unpaired Student t test (GraphPad Software, La Jolla, California, United States). *P* values of <0.05 were considered statistically significant.

## Results

**Fig. 1:**
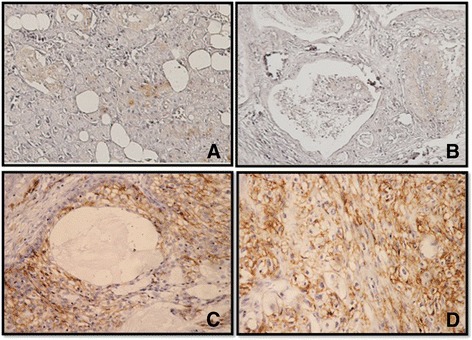
Immunohistochemistry for CD44 in Mucoepidermoid carcinomas. **a** Normal salivary glands showed weak cytoplasmic stain in ductal epithelium and no immunoreactivity is seen in acinic cells. **b** No or mild CD44 expression was observed in the membrane of mucoepidermoid carcinoma cells (low grade type). **c**, **d** CD44 expression was strongly expressed in the membrane of the mucoepidermoid carcinoma cells (intermediate and high grade type)

The mean age of the 15 patients was 49.7 years (range: 21–70). There were nine (60 %) female and six (40 %) male patients. There were 13 cases from the parotid gland and two from the submandibular gland. Five cases (33.3 %) were classified as low-grade, two cases (13.3 %) as intermediate-grade and eight (53.3 %) as high-grade tumors. Regional lymph node metastasis was noted in eight cases (53.3 %), distant metastases were present in four cases (26.7 %), and local recurrence was found in four patients (26.7 %). Immunohistochemical tissue staining was performed to detect the expression of CD44 in normal salivary glands and in MEC tissue. Representative results are shown in Fig. 1. CD44 expression was found in 13 cases (86.7 %) and considered weak in two (13.3 %), moderate in three (20 %) and strong in eight (53.3 %) cases. Membranous staining was seen in mucous, intermediate, myoepithelial and epidermoid cells in tumor tissues while in normal tissue, staining was positive in lymphocytes, while weak focal cytoplasmic staining was observed in ductal cells. Data on CD44 expression, histological grade, lymph node involvement, recurrence and metastasis are summarized in Table 1. High-grade tumors showed moderate to strong CD44 expression (87.5 %) more frequently than did low-grade tumors (40 %), but this difference was not statistically significant (*P* =0.2108). CD44 expression was strong in tumors of patients who presented local recurrence and metastasis around 75 % in each group, although this difference in expression compared to tumors of patients who did not have local recurrence or metastasis is failed to meet statistical significant (*P* = 0.5327, 0.2839). No correlation was found between CD44 expression and lymph node involvement (*P* = 0.3599; *P* < 0.05). Survival data were not available and not included in this study.

**Table 1:** CD44 expression according to histological grade of mucoepidermoid carcinoma cases

		Location	CD44 Expression			
		*P/S 13/2	Negative (%)	Weak (%)	Moderate (%)	Strong (%)
Grade						
Low grade		5/0	2/5 (40)	1/5 (20)	1/5 (20)	1/5 (20)
Intermediate grade		1/1	0/2 (0)	0/2 (0)	1/2 (50)	1/2 (50)
High grade		7/1	0/8 (0)	1/8 (12.5)	1/8 (12.5)	6/8 (75)
Lymph node						
Positive	8/15	7/2	0/8 (0)	1/8 (12.5)	2/8 (25)	5/8 (62.5)
Negative	7/15	7/0	2/7 (28.6)	1/7 (14.3)	1/7 (14.3)	3/7 (42.85)
Metastasis	4/15	4/0	0/4 (0)	1/4 (25)	0/4 (0)	3/4 (75)
Recurrence	4/15	3/1	0/4 (0)	0/4 (0)	1/4 (25)	3/4 (75)

*P; Parotid gland. S; Submandibular gland

## Discussion

Mucoepidermoid carcinoma shows widely diverse biological behaviors. High-grade MEC is a highly aggressive tumor, whereas low-grade MEC shows a more benign nature [15]. In our study, MEC was more frequent in the parotid gland of female patients, primarily between the third and sixth decades of life. Molecular alterations associated with histologic and clinical behavior of this tumor are still not clearly understood, and therefore additional studies are necessary. In this study, we examined the expression of CD44 in normal salivary gland and in MEC tissues of different histological grades. CD44 is an adhesion molecule and a marker of CSCs that is expressed in several normal and tumor tissues. Metastasis and aggressiveness have been correlated with CD44 expression in breast cancer and renal cell carcinoma [5]. Our immunohistochemistry analysis showed that CD44 expression is substantially upregulated in MEC tissues compared with adjacent normal tissue, indicating that CD44 may contribute to tumorigenicity in MEC, but we failed to observe statistically significant differences in CD44 expression in recurring and metastasized MEC as compared to other MEC. Chang et al. found that the expression of HA but not its receptors CD44 and the hyaluronan receptor for endocytosis (HARE) are associated with MEC metastasis and lymph node involvement [16]. Xing et al. found that HA and CD44 are expressed in most malignant salivary gland tumors including MEC [17].

Analysis of the expression of CD44 and its variant isoforms in salivary gland tumors has yielded different results depending on the type of tumor studied. Fok et al. showed low expression of CD44 in salivary gland tumors (pleomorphic adenoma, polymorphous low grade adenocarcinoma and adenoid cystic carcinoma) compared with normal tissue [18]. This result is consistent with our data showing that low expression of CD44 is associated with less aggressive tumors. Wein et al. reported expression of HA and its associated receptors CD44 and HARE in mucoepidermoid carcinoma [19]. Other studies using qPCR support these findings; CD44 was overexpressed in the tumors from salivary glands compared with normal salivary glands [20]. CD44v3 and v6 variants are widely expressed by the myoepithelial cells of salivary gland tumors and are correlated with the ability to self-renew [21].

We noted a tendency of higher CD44 expression in high-grade tumors compared to low grade, so it can serve as tumor behavior indicator. Since the numbers of cases of each grade of mucoepidermoid carcinoma investigated in this study were relatively small, the relationship between clinicopathological factors and CD44 expression levels in each grade of carcinomas could not be clarified, however, we found that the CD44 staining is varies among different stage of MEC. Another limitation of our study is the incompleteness of patient records, including lack of stage and survival data, which could limit our results.

The mechanism by which a tumor cell invades the surrounding structure is poorly understood in MEC. Several studies suggest that extracellular matrix composition might regulate cell invasion, and that HA and CD44 might facilitate the invasive behavior of tumors [7]. In conclusion, increased expression of CD44 might play an important role in increasing the potential for tumorigenesis; as well as, we found a trend from our results that the expression of this molecule can be employed as a predictor of tumor behavior and recurrence but further studies with other markers and bigger samples may be more helpful in this regard.

## Conclusion

CD44 showed to be expressed by neoplastic cells in MEC like other type of cancer. This expression was not found to correlate with the lymph node involvement of tumor. We observed that CD44 expression was associated with four metastasis cases and four recurrent cases but no significant relation was found and this indicated that CD44 is not a prognostic indicator. In other hand, our study showed a differential staining of CD44 in MEC in different histological grades and this may be an interesting finding that should be explored further in other study with actual correlation with prognosis.

## Abbreviations

AFIP: Armed Forces Institute of Pathology
CSCs: cancer stem cells
HA: hyaluronan
HARE: hyaluronan receptor for endocytosis
MEC: Mucoepidermoid carcinoma
